# Monthly Reports

**Published:** 1803-02-01

**Authors:** J. Ricards

**Affiliations:** 68, Hutton Street


### MONTHLY REPORTS.

*Cases admitted under the Care of the Surgeon of the Finsbury Dispensary, St. John's Square, Clerkenwell, from December* 10, 1802, *to January* 10, 1803.

| | | | |
|---|---|---|---|
| Phlegmone Genu | 1 | Leucoma | 1 |
| Ophthalmia | 1 | Vulnera | 3 |
| Erysipelas | 4 | Contusiones | 3 |
| Mastodynia | 2 | Combustura | 5 |
| Abscessus Axillæ | 1 | Lues | 2 |
| ——— Perinæi | 1 | Schrophula | 2 |
| ——— Genu | 1 | Hydrarthus Genu | 1 |
| ——— Scrophulosi | 4 | Hernia Ventralis | 1 |
| Paronychiæ | 2 | Hydrocele | 1 |
| Furunculus | 1 | Odema Scroti | 1 |
| Mortificationes Cruris | 2 | Tinea | 1 |
| Ulcera Cruris | 14 | Eruptiones Chronicæ | 4 |
| ——— Capitis | 2 | | — |
| Ulcus Palati | 1 | Total | 62 |

The diseases of the last month, as will appear by the above Report, have been of a very miscellaneous nature; no particular epidemic (in the department of Surgery) having occurred to occupy the attention.

The erysipelatous affections, which we mentioned in the last Report to have been so extremely frequent, and attended with so much malignancy of symptoms, have appeared but in a very few cases since that time; which cases were of a much milder nature, creating little or no alarm.

The temporary frost has, in several instances, been the occasional cause of mortification in the extremities of aged and infirm persons.

The most distressing disease which occurs among the poorer classes of society, and which is productive of most trouble to the surgeon, is that of strictures in the urethra, advanced to such a degree of obstruction as to have produced abscesses and consequent fistulæ in the perinæum or adjoining parts. These are cases which occur comparatively very frequently, as it may be perceived scarcely a month passes that does not produce one or more cases at this Dispensary. The frequency of the disease seems to proceed from the mode by which the laborious order of mankind generally treat the first appearance of simple gonorrhœa. There are in most neighbourhoods, or within a certain circle of connexion, one or more men (common labourers) who have a specific for the disease, and who are therefore applied to by their brethren when in want of relief, relief. The remedy is generally an injection of a stimulating nature, which is, of course, indiscriminately used in every stage or variety of the complaint. Or, application is made to those shops where "injection powders" are advertized in the windows. The superior order of Empirics, whose nostrums can seldom be procured under half a crown, are the bane of a class of society one degree superior to those to whom we advert, who seldom, in the first instance, advance so high in their purchase of a remedy.

It must be evident that the indiscriminate use of these applications, which are, for the most part, compositions of white vitriol, blue vitriol, or corrosive sublimate, all of which are excellent remedies when properly adapted, produce such a state of the urethra, as to lay the foundation for strictures. When a stricture has formed, and a difficulty occurs in voiding the urine, the symptoms are attributed to gravel; and turpentine, and other stimulants of a like nature, with a copious supply of gin, are swallowed, "to force the water;" or, in consequence of the continuing gleet, the disease is considered as a weakness, and the original cause of the disease, stimulating injections, are still more assiduously applied; this latter error is sometimes fallen into, even by regular practitioners. If at this time application is made to a surgeon, who perceives the nature of the case, it very seldom happens, that persons in this sphere of life can or will adhere for a sufficient length of time, and with sufficient diligence, to the plan prescribed; it has been frequently observed to me, by persons of this description, that they had had a bougie introduced *two or three times*, but as they had experienced no relief, they had desisted from the attempt. Thus the disease acquires inveteracy, till at length the urethra gives way, the urine escapes, and abscesses and their consequences are the result. I have a patient at this time, where disease has followed a course very similar to this description; he had, fourteen years ago, a gonorrhœa, which was not attended to by any regular practitioner, but treated with nostrum injections; the gonorrhœa yielded, but after a short time he experienced some difficulty of micturition, which after suffering for some time, became the subject of consultation with a regular surgeon; this gentleman very properly recommended a continued use of bougies, and introduced them once or twice; when the patient, not finding immediate relief discontinued his attendance. For several years his disease gradually became worse, nor did he profit himself by further surgical assistance, till he became my patient, which was was in consequence of an abscess forming in the perinæum; the whole of the bottom of the abdomen, likewise, the scrotum, &c. &c. were in a most violent and extensive state of inflammation, from the effusion of urine; the abscess in the perinæum I opened deeply, whereby the urine obtained a free passage; but not before it had created so much mischief, as to produce an extensive sloughing of the parietes of the abdomen, scrotum, and integuments of the penis.

The innovation in the treatment of strictures which has very much obtained within these five years, from the authority of Mr. Home, who has improved upon the mode recommended by Mr. John Hunter; by the use of caustic applied by means of bougies, instead of those bougies which act merely mechanically, is an improvement in Surgery of great importance, when confined within its proper limits, and adapted to those particular cases to which it is peculiarly appropriate. Two cases, wherein their beneficial effects were particularly apparent, and well delineated, (besides many others of a less formidable appearance) have recently occurred in my practice; both these cases were fistula. In one of them, the aperture was over the pubis; in the other, there were several apertures in the perinæum and scrotum; in both, very little urine passed except by the fistula; in neither could the smallest bougie be passed during several weeks assiduous attempt; at length I had recourse to the caustic. After a few applications the urethra became pervious, and by a continued use of the common bougie for some little time, the disease was cured. The fistula healed by the assistance only of a stimulating injection, and no inconvenience was sustained.

But while I record these fortunate cases, I must confess that many instances have come to my knowledge, wherein much important injury has been sustained by the use of the caustic bougies. I am well convinced that of late their use has been too extensive: valuable remedies are generally active remedies; and active remedies, when indiscriminately used, will be productive, in the aggregate, of more harm than good; in my opinion, although this practice is an improvement of considerable consequence, its use should not be adopted till sufficient trial has proved the common mode ineffectual, but as a dernier resort when all other remedies have failed.

68, *Hatton Street.* J. RICARDS.

